# Intrapartum fever complicated with maternal bacteremia: prevalence, bacteriology, and risk factors

**DOI:** 10.1007/s00404-024-07564-5

**Published:** 2024-05-26

**Authors:** Emmanuel Attali, Guy Kern, Miriam Warshaviak, Lee Reicher, Ariel Many, Yariv Yogev, Yuval Fouks

**Affiliations:** 1 Department of Obstetrics and Gynecology, Lis Hospital for Women, Tel Aviv Sourasky Medical Center, Weizman 6, Tel Aviv, Israel; 2 Faculty of Medicine, Tel Aviv University, Tel Aviv, Israel; 3 Boston IVF, 130 2nd Ave, Waltham, MA 02451 USA

**Keywords:** Intrapartum fever, Bacteremia, Antibiotics

## Abstract

**Purpose:**

To assess the prevalence, microbial profile, and clinical risk factors of maternal bacteremia associated with intrapartum fever (IPF).

**Methods:**

A retrospective cohort study, in a single tertiary university-affiliated medical center between 2012 and 2018. Demographic and labor characteristics of women, who delivered at term (37+0/7–41+6/7) and developed bacteremia following IPF were compared to a control group of women with IPF but without bacteremia.

**Results:**

During the study period there were 86,590 deliveries in our center. Of them, 2074 women (2.4%) were diagnosed with IPF, of them, for 2052 women (98.93%) the blood maternal cultures were available. In 26 patients (1.25%) maternal bacteremia was diagnosed. A lower rate of epidural anesthesia (84.6% vs 95.9%, *p* = 0.02) and a higher rate of antibiotics prophylaxis treatment prior to the onset of fever (30.8%.vs 12.1%, *p* = 0.006) were observed in patients who developed maternal bacteremia in comparison to those who have not. Maternal hyperpyrexia developed after initiation of antibiotics or without epidural anesthesia remained significantly associated with maternal bacteremia after applying a multivariate analysis, (Odds Ratio 3.14 95% CI 1.27–7.14, *p* = 0.009; 4.76 95% CI 1.35–12.5, *p* = 0.006; respectively).

**Conclusion:**

Maternal fever developing after initiation of antibiotics or without epidural is associated with maternal bacteremia.

## What does this study add to clinical work?

**Table:** 

The study was created to assess the correlation between intrapartum fever and maternal bacteremia and to estimate the extent of antimicrobial overuse in cases of intrapartum fever.
Maternal bacteremia was found in roughly 1.5% of total cases of intrapartum fever at term. Two major risk factors for maternal bacteremia were found: the development of fever despite the prior initiation of an antimicrobial regimen for GBS prophylaxis and intrapartum fever in women without an epidural.

## Introduction

Intrapartum fever (IPF) is defined as the maternal temperature measured during labor that is equal to or above 38 °C and IPF complicates approximately 5% of all deliveries [1, 2]. Maternal intrapartum fever can lead to various maternal and neonatal complications. Moreover, intrapartum fever may influence long-term offspring susceptibility to pediatric infections [3].

Typically, IPF reflects a sequence of physiologic responses involving several mediators which are a mere part of the process of physiologic labor rather than an underlying infectious condition [4, 5]. Factors such as epidural anesthesia, prolonged labor, premature rupture of membranes (PROM), and induction of labor [6] are all sought to have a pyrogenic trajectory and therefore have been identified as risk factors for IPF.

The underlying fetal or maternal infectious process in intrapartum fever was a focus of only some amount of research principally assessing short and long-term neonatal adverse outcomes [1]. However, data are limited regarding IPF and its association with maternal bacteremia. Prior studies that investigated maternal bacteremia included relatively small sample sizes [7, 8] and utilized a broader timeframe which included the peripartum period [7, 8]. Those studies were not able to identify the specific pathogens involved in intrapartum fever due to small sample sizes and the inclusion of prepartum and post-partum cases.

In this study, we aim to assess the prevalence, microbial profile, and clinical risk factors of maternal bacteremia associated with intrapartum fever.

## Material and methods

### Subjects and study design

We performed a retrospective cohort study, in a single tertiary university-affiliated medical center between January 1, 2012, and December 31, 2018.

This study was approved by the Medical Center Review Board (IRB). IRB approval number: 0284-08. Due to the retrospective nature of the study, informed consent was not required.

We extracted medical charts of patients at term (37^+0^–41^+6^ weeks of gestation) with intrapartum fever, defined as the orally measured temperature above ≥ 38.0 °C at least once during labor and before delivery. According to our departmental protocol, four bacterial cultures were obtained from peripheral veins in any case of intrapartum fever and prior to initiation of antibiotics treatment other than GBS prophylaxis [2]. (Known GBS carrier women were not excluded. In Israel, there is still no policy for screening and the decision to screen is to the discretion of the physician, usually between weeks 35–37. The incidence of GBS carrier is 15–16% [9]).

Electronic medical files were cross-referenced with a microbiologic registry which included blood culture results and susceptibility testing.

Data including patient demographics, relevant medical history, obstetrics, and labor characteristics (Group* B Streptococcus* (GBS) status, the time interval from membrane rupture to delivery; the presence of meconium; type of anesthesia; mode of delivery, as well as laboratory data (maximal white blood cell count during labor) were collected. Previous hospitalizations, prior hospital-acquired infections, and known maternal risk factors for infection (diabetes, obesity, immune-compromised states, and smoking) were identified.

The blood cultures were analyzed within 48 h. The bacterial identification and antibiotic susceptibility test results were performed at the local microbiology laboratory using the Vitek2 system (bioMerieux, St. Louis, Mo) according to the clinical and laboratory standards institute (CLSI) criteria [10, 11].

The study group included all women who developed intrapartum fever and bacteremia with true positive blood cultures. In contrast, the control group was comprised of women who developed intrapartum fever and the blood cultures stayed sterile or positive for contaminants only.

To define a true positive blood culture, we eliminated cases of potential contamination after consultation with an infectious disease specialist. Women with only one blood culture positive for contaminants defined as *coagulase-negative Staphylococcus, Bacillus species, Propionibacterium, Veillonella, Micrococcus, and Corynebacterium species,* were excluded from the analysis. Organisms were classified as contaminants based on discussion with the microbiological laboratory and our infectious disease consultant.

### Statistical analysis

Patients’ characteristics were summarized with descriptive statistics. Mean (standard deviation, SD) and median (interquartile range, IQR) were used for the description of normally and non-normally distributed quantitative variables, respectively. Distribution normality was determined using histograms. Normally distributed values were compared using independent samples Student’s *t* test while the Mann–Whitney test was utilized for non-normally distributed covariates. Fisher’s exact test was performed on all categorical variables.

Univariable analysis was performed to assess candidate variables as risk factors for maternal bacteremia. The associations between potential risk factors and the outcome were quantified by the OR and 95% confidence interval (CI). Major maternal leukocytosis was defined as maximal white blood cell count over 30,000 cells/microliter [12], and severe pyrexia a measured temperature was ≥ 39.0 °C [7]. Multivariable forward stepwise logistic regression was performed to assess the relationship between clinical characteristics and positive blood cultures. Variables were selected as candidates for the multivariable analysis based on the level of significance of the bivariate association (*p* < 0.1). Missing data were handled using list-wise deletion after assuming for missing at random. All the available data were used for the generation of graphs. Data analysis was conducted with Statistical Package for the Social Sciences, version 25.0 (IBM SPSS Statistics for Windows, Version 25.0. Armonk, NY: IBM Corp) and Microsoft Excel version 14.0 (Microsoft Corporation, Redmond, Washington).

## Results

**Fig. 1:**
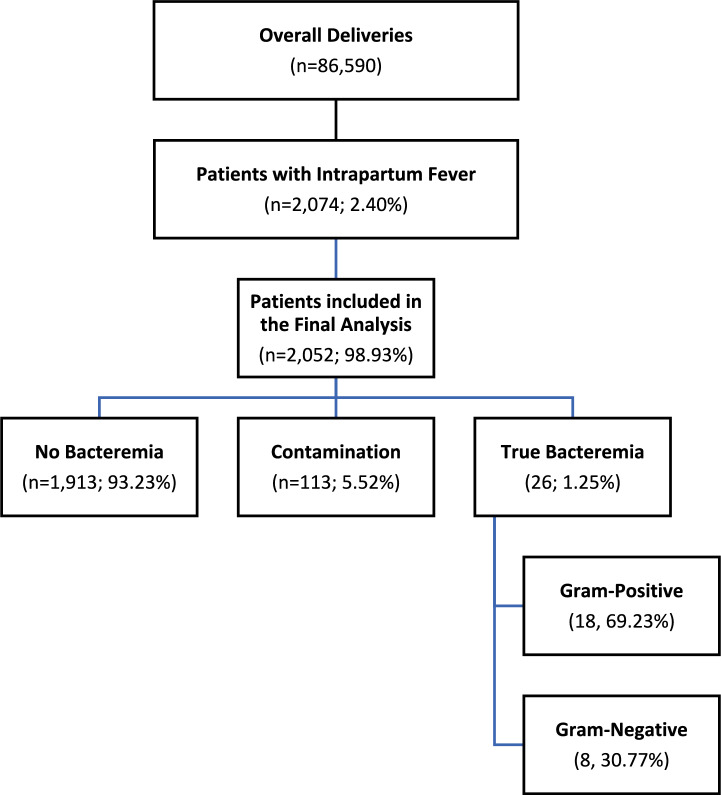
Flow chart

During the study period, there were 86,590 attempted vaginal deliveries in our center. The final study cohort is presented in Fig. 1. Overall, 2074 women (2.40%) developed IPF. The results of 2052 (98.93%) maternal blood cultures were available for analysis. Of these, 1823 (88.84%) cultures set resulted negative. In addition, 113 (5.51%) blood culture sets were positive for known contaminants such *as Staphylococcus Coagulase-Negative, Micrococcus, Diphtheroid, and Bacillus species.* and were excluded.

**Fig. 2:**
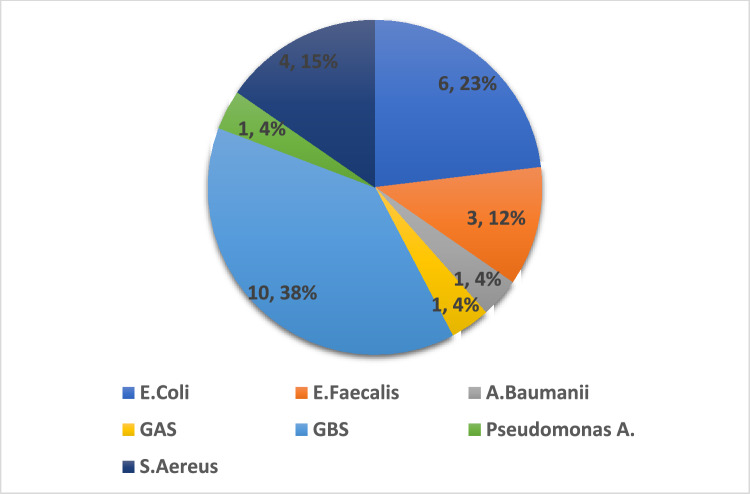
Bacteriology

In 26 patients (1.25%), true maternal bacteremia was diagnosed. The bacteriological characteristics of isolated organisms are presented in Fig. 2.

The majority of pathogens were gram-positive organisms (69.23%), of which the most common was *Group B Streptococcus* (10 cases). Notably, four of the women who presented with GBS sepsis were screened and found positive in the late third trimester and were treated by antimicrobial prophylaxis, four other patients had an unknown GBS status and two were screened for the pathogen and were found to be negative.

Gram-negative bacteria accounted for less than a third of the cases of bacteremia (30.77%), the most common pathogen of which was *Escherichia Coli*. Five of six women were diagnosed with an *Escherichia Coli-associated* urinary tract infection*.*

All the pathogens were sensitive to the empiric regimen given at the time that the IPF was diagnosed. There were no multi-resistant strains in the blood samples analyzed in our cohort.

Demographic and clinical characteristics of women with and without bacteremia are presented in Table 1. Patients with epidural anesthesia had a lower risk of having bacteremia (80.6% vs 95.2%, *p* = 0.004). Moreover, patients with bacteremia had higher rates of antibiotic treatment for GBS prophylaxis prior to the development of fever (30.8%.vs 12.1%, *p* = 0.006).

**Table 1:** Maternal and obstetrical characteristics

	No bacteremia (*N* = 2052)	Maternal bacteremia (*N* = 26)	*p* value
Maternal age, years [IQR]	31 [28–34]	31 [28–35]	0.67
Gestational age, weeks**,** [IQR]	40.0 [39–41]	39.5 [39–41]	0.75
Postdate pregnancies, *n* (%)	1151 (56.1)	13 (50)	0.15
Primigravidae, *n* (%)	1702 (83.0)	20 (76.9)	0.43
Previous cesarean delivery, *n* (%)	112 (5.5)	0	–
Gestational diabetes mellitus, *n* (%)	159 (7.7)	2 (7.7)	0.61
Hypertensive disorders of pregnancy, *n* (%)	348 (17.0)	5 (19.2)	0.97
Epidural anesthesia, *n* (%)	1967 (95.9)	22 (84.6)	0.022
Mode of delivery, *n* (%)			
Normal vaginal delivery	1202 (58.6)	13 (50.0)	0.61
Operative vaginal delivery	444 (21.6)	7 (26.9)	
Cesarean delivery	406 (19.8)	6 (23.1)	
GBS carrier, *n* (%)	332 (16.2)	5 (19.2)	0.60
Meconium stained amniotic fluid, *n *(%)	437 (21.3)	3 (11.5)	0.412
Antibiotics treatment prior to fever, *n* (%)	249 (12.1)	8 (30.8)	0.01
Max WBC 10^3^/microliter, [IQR]	18.2 [15.2–21.6]	19.2 [15.2–25.3]	0.24
Max temperature, C, [IQR]	38.3 [38.1–38.5]	38.5 [38.3–38.9]	0.006
Systolic blood pressure, [IQR]	131 [123.0–138.0]	129.5 [122.8–139.0]	0.83
Pulse, [IQR]	102 [92–113]	110 [94–119]	0.20

Data are presented as mean; ± standard deviation

*GBS* Group B Streptococcus, *WBC* white blood cell

No differences were found regarding maternal age at delivery, gestational age at delivery, the time interval from rupture of the membrane to delivery, rate of meconium-stained amniotic fluid, the prevalence of gestational diabetes, rate of *Group B Streptococcus* colonization in the third trimester at routine screening or mode of delivery (Table 1).

The epidural rate was lower in the study group (84.6% vs 95.9%, *p* = 0.22). The mean maximum temperature measured was slightly higher in the study group (38.5 [0.6] vs 38.3 [0.4], *p* = 0.006) and more women were treated with antibiotics prophylaxis before the onset of the fever (30.8% vs 12.1%, *p* = 0.01). No differences were found in mean maximal White Blood Cells (WBC), nor in vital signs (systolic and diastolic blood pressure, and pulse rate) (Table 1).

No difference was found in short-term neonatal outcomes between the groups (Table 2) including birth weight, Apgar score under 7 at 5 min, and admission rate to the neonatal intensive care unit. There was one case of reported perinatal death associated with a fulminant *GBS* sepsis proven by the newborn blood culture. The infection was related to vertical transmission of GBS as the mother was a known carrier. She was admitted with a premature rupture of membrane and was treated by Penicillin for GBS prophylaxis but developed an intrapartum fever. The antibiotic treatment was switched to Gentamicin and Ampicillin. The newborn was delivered 15 h following the rupture of membrane, Apgar score of 5 and 9, and was transferred to Neonatal Intensive Care Unit. He underwent a full sepsis workup, and GBS was isolated from blood culture. He deceased 2 days later despite antibiotics treatment.

**Table 2:** Neonatal characteristics

	No bacteremia (*N* = 2052)	Maternal bacteremia (*N* = 26)	*p* value
Birthweight, gr [IQR]	3362 [3090–3640]	3217 [3003–3414]	0.07
Birthweight < 2500 gr *n* (%)	35 (1.71)	0	–
Birthweight > 4000 gr, *n* (%)	118 (5.75)	0	–
Apgar under 7 at 5 min, *n* (%)	22 (1.1)	0	–
Admission to NICU, *n* (%)	93 (4.5)	4 (15.4)	0.08
Max WBC Value, 10^3^/microliter, [IQR]	22.8 [18.7–27.5]	22.1 [15.8–29.0]	0.42

Data are presented as mean; ± standard deviation

*WBC* white blood cell

A univariate logistic regression identified risk factors associated to maternal bacteremia: intrapartum fever above 39.0 °C (Odd Ratio 3.11 95% CI 1.03–7.75, *p* = 0.025); WBC count above 30,000 cells/microliter (Odd Ratio 4.30 95% CI 1.00–12.79, *p* = 0.020); fever developed after initiation of antibiotics (Odd Ratio 3.22 95% CI 1.31–7.25, *p* = 0.007) and maternal pyrexia without epidural anesthesia (adjusted Odd Ratio 4.35 95% CI 1.21–11.11, *p* = 0.025) (Table 3).

**Table 3:** Odds Ratio (OR) for maternal bacteremia

	OR	OR
	Univariable analysis	Multivariable analysis
Fever^b^	**3.11** 95% CI 1.03–7.75, *p* = 0.025	**2.81** 95% CI 0.92–7.13, *p* = 0.044
Leukocytosis^a^	**4.30** 95% CI 1.00–12.79, *p* = 0.020	**3.84** 95% CI 0.88–11.70, *p* = 0.035
Prior Antibiotic Treatment	**3.22** 95% CI 1.31–7.25, *p* = 0.007	**3.14** 95% CI 1.27–7.14, *p* = 0.009
Labor without Epidural Anesthesia	**4.35** 95% CI 1.21–11.11, *p* = 0.025	**4.76** 95% CI 1.35–12.5, *p* = 0.006

Bold indicates the OR

^a^Leukocytosis is defined as a maximal White blood cell value over 30,000 cells/microliter

^b^Intrapartum fever is defined as orally measured temperature ≥ 39.0 °C at least once during labor

After multivariate analysis with adjustment to antepartum and intrapartum factors (including antibiotics treatment prophylaxis, maximal maternal fever, leukocytosis, and epidural anesthesia), intrapartum fever above 39.0 °C and maternal leukocytosis lost their statistical significance (adjusted Odd Ratio 2.81 95% CI 0.92–7.13, *p* = 0.044; 3.84 95% CI 0.88–11.70, *p* = 0.035; respectively). Nevertheless, fever developed after initiation of antibiotics or without epidural anesthesia remained a significant and independent risk factor for the development of maternal bacteremia (Odd Ratio 3.14 95% CI 1.27–7.14, *p* = 0.009; 4.76 95% CI 1.35–12.5, *p* = 0.006; respectively).

## Discussion

### Principal findings

In this study, we aimed to assess the prevalence, microbial profile, and clinical risk factors of maternal bacteremia associated with intrapartum fever We have concluded that: (1) the rate of true maternal bacteremia in the setting of intrapartum fever is low and accounts for 1.25% of cases; (2) more than 2/3 of the pathogens accounted as causative agents to maternal bacteremia are Gram-positive, with streptococcus agalactia as most common; and (3) the development of fever after initiation of prophylaxis or without epidural anesthesia are independent risk factors for maternal bacteremia.

### Results of the study in the context of other observations

In a previous study by Wilkie et al. [8], the rate of maternal bacteremia was reported to be lower, and the microbiological characteristics of the isolated patterns were also slightly different. In their study, the most common pathogen was *Escherichia Coli* and accounted for 18% of all isolated organisms. In our research, the latter was found in 23% of cultures while the most common pathogen was *Group B Streptococcus* (38%). Notably, the two studies differ by population; while Wilkie and colleagues included women with a fever that occurred between 7 days before and up to 30 days after the delivery, we focused on patients with intrapartum fever, reflecting a current debate regarding the latter event as a prodrome of true infection.

A case-control study by Easter et al. [7] assessed risk factors associated with maternal bacteremia in febrile peripartum women and established that both the initial fever during labor and the presence of fever at or above 102 °F (38.9 °C) were independent risk factors for the development of bacteremia. In our study, after removing potential confounders to antepartum and intrapartum factors, intrapartum fever above 39.0 °C lost its statistical significance but a trend was noticed.

The impact of an isolated maternal fever is described by the ACOG Committee Opinion [13] as the maternal temperature between 38.0 and 38.9 °C with no additional risk factors present is still under debate. A recent publication by Bank et al. [14], reported a lower rate of treatment of endometritis among women who were treated with antibiotics for this indication. However, they noticed a lower 5-min Apgar score in comparison to women with isolated intrapartum fever who were not treated. The rate of maternal bacteremia following isolated intrapartum fever was not reported. In our study, 80.7% of maternal bacteremia occurred when the reported maximal temperature was under 39.0 °C. Moreover, although the mean maximum temperature measured was statistically slightly higher in the group of women who developed bacteremia, this change is not of clinical significance.

Similarly, a high leukocyte count (above 30,000 cells/microliter) lost its statistical significance after adjustment for covariates and 88.5% of maternal bacteremia occurred while the reported maximal WBC count was under 30,000 cells/microliter. Conversely, in a previous recent study [15], we reported that a high WBC in preterm delivery was an independent risk factor for maternal bacteremia. In the same study, we found that preterm meconium was also associated with maternal bacteremia. This association was not found in our present study.

The only independent risk factors associated with intrapartum maternal bacteremia were fever that developed after initiation of antibiotics and maternal pyrexia that occurred without epidural anesthesia.

### Clinical implications

As far as we know, this is the first study that evaluates the significance of intrapartum fever occurring after the initiation of antibiotics for GBS prophylaxis. In contrast, the association between epidural anesthesia and maternal pyrexia is well-known and studied [4, 16]. However, the association of epidural anesthesia-induced fever with bacteremia is not clear. The authors of a systemic review and meta-analysis on epidural-related fever and maternal neonatal morbidity published in 2020 [17] called for further research on whether epidural-related intrapartum fever is associated with an infectious process. A recent study by Ward and Caughey [18] examined whether intrapartum fever carried the same risk for neonatal sepsis with and without an epidural. They found a decreased risk of neonatal sepsis in the setting of intrapartum fever with an epidural (aOR 0.53 [95% CL 0.29–0.98]).

Our study provides important insights into the incidence of maternal bacteremia in women with intra-amniotic infection and the risk factors associated with its development. The overall rate of maternal bacteremia was found to be 1.25%, which is consistent with some previous studies. The observed lower rate of epidural anesthesia and a higher rate of antibiotic prophylaxis treatment prior to the onset of fever in women with maternal bacteremia suggest that these factors may play a noncausal role in the development of maternal bacteremia. In addition, the finding that maternal hyperpyrexia after initiation of antibiotics or without epidural anesthesia was significantly associated with maternal bacteremia indicates that these factors may be important in identifying women at higher risk of developing bacteremia.

These results have important clinical implications, but since the numbers of adverse outcomes (absolute risk) are so low, it cannot simply infer that careful monitoring of maternal temperature and judicious use of epidural anesthesia and antibiotics prophylaxis help reduce the crude incidence of maternal bacteremia in a measurable way. Further studies are needed to confirm these findings and identify additional risk factors that may contribute to the development of maternal bacteremia. Nonetheless, the present study provides a valuable contribution to our understanding of the risk factors associated with maternal bacteremia in women with intra-amniotic infection and highlights the importance of timely identification and management of this condition.

### Strengths and limitations

The strengths of our study include its large sample size which allows an analysis of risk factors for a rare outcome under a similar and relatively homogenous treatment policy. In addition, the single-center design of the study enabled uniform policy and protocols.

As far as we know, it is the first study to estimate risks and elaborate on the true microbiological profile of pathogens in this potentially severe complication at term pregnancy.

However, our study is limited mainly due to its retrospective nature and lack of information such as the time of first-documented fever, time from epidural to fever, data on C-Reactive Protein, and classification of hypertensive disorders of pregnancy. Similarly, long-term maternal and neonatal outcomes are unavailable. An additional limitation is that anaerobic bacteria were underestimated in our study as they were only partially reported due to local laboratory practices of collection methods. While these pathogens are targeted in all of the antimicrobial regimens detailed, their role as causative pathogens in the context of maternal bacteremia is questioned.

## Conclusion

The results indicate that the rate of true maternal bacteremia in the setting of intrapartum fever is low, with more than 2/3 of causative agents being Gram-positive, and the development of fever after initiation of prophylaxis or without epidural anesthesia are independent risk factors for maternal bacteremia. The study also highlights that the association between epidural anesthesia-induced fever and bacteremia is not clear. However, routine blood culture and prolonged antibiotics treatment should be recommended for women who develop fever under antibiotics prophylaxis or without epidural anesthesia.
